# Causal relationship between sarcopenia and rotator cuff tears: a Mendelian randomization study

**DOI:** 10.3389/fendo.2024.1436203

**Published:** 2024-10-29

**Authors:** Dongmei Yang, Zheng Li, Ziqing Jiang, Xianzhong Mei, Daguang Zhang, Qiushi Wei

**Affiliations:** ^1^ Department of Orthopedics, Shenzhen Pingle Orthopedics Hospital(Pingshan District Hospital of Traditional Chinese Medicine, Shenzhen, Guangdong, China; ^2^ The Third Clinical Medical College, Guangzhou University of Chinese Medicine, Guangzhou, Guangdong, China; ^3^ School of Traditional Chinese Medicine, Southern Medical University, Guangzhou, Guangdong, China; ^4^ Department of Orthopedics, The First Bethune Hospital of Jilin University, Changchun, Jilin, China; ^5^ Traumatology & Orthopaedics Institute, Guangzhou University of Chinese Medicine, Guangzhou, Guangdong, China; ^6^ Department of Orthopedics, The Third Affiliated Hospital, Guangzhou University of Traditional Chinese Medicine, Guangzhou, Guangdong, China

**Keywords:** causal relationship, rotator cuff tears, sarcopenia, genetic epidemiology, skeletal muscle disease

## Abstract

**Background:**

Sarcopenia and rotator cuff tears are common among elderly patients. However, the role of sarcopenia in the management of rotator cuff tears has been often overlooked. This study aimed to elucidate the effects of sarcopenia-related traits on rotator cuff tears.

**Methods:**

Two-sample Mendelian randomization (MR) analyses based on genome-wide association study data were used to evaluate the causal relationships among appendicular lean mass (ALM), usual walking pace, low hand grip strength, and rotator cuff tears. Multivariate Mendelian randomization (MVMR) analyses were used to evaluate the direct effects of each muscle trait on the causal relationship.

**Results:**

Univariate MR analysis showed that ALM and usual walking pace were causally related to rotator cuff tears (odds ratio (OR) = 0.895; 95% confidence interval (CI), 0.758-0.966, *P*<0.001 and OR = 0.458, 95% CI, 0.276-0.762, *P* = 0.003, respectively), and there was no evidence of causality between low hand grip strength and rotator cuff tears (OR = 1.132, 95% CI, 0.913-1.404, *P* = 0.26). MVMR analysis confirmed the causal effects of ALM and walking pace on rotator cuff tears (OR = 0.918, 95% CI, 0.851-0.990, *P* = 0.03 and OR = 0.476, 95% CI, 0.304-0.746, *P* = 0.001, respectively).

**Conclusion:**

A causal genetic relationship exists between sarcopenia and rotator cuff tears. Sarcopenia-related traits including low muscle mass and physical function, increase the risk of rotator cuff tears. These findings provide new clinical insights and evidence-based medicine to optimize management of rotator cuff tears.

## 1 Introduction

The rotator cuff is an important dynamic anatomical complex that maintains shoulder stability and provides precise spatial positional control to achieve shoulder torque balance. Therefore, it is prone to injury during daily activities and upper limb movements, resulting in varying degrees of pain and functional impairment, which seriously affect patients’ quality of life. Rotator cuff tears account for 13–41% of shoulder disorders (1, 2) and have a linear correlation with ageing, with a morbidity rate of 13% in people aged >50 years and 20% for those aged >60 years (3, 4). With an increasingly ageing population worldwide, rotator cuff diseases have become a progressively serious social health problem with enormous medical expenses (5). Early identification of related risk factors and timely interventions have an important clinical value for the management of rotator cuff tears. Previous studies have shown that the occurrence of rotator cuff disease is accompanied by tendon cell atrophy and fat infiltration, with some genetic susceptibility (6).

Sarcopenia is a geriatric syndrome characterized by age-related loss of skeletal muscle mass, muscle strength, or physical function (7). It is estimated that approximately 50 million people worldwide suffer from this condition, with a prevalence rate of 10–27% in patients aged >60 years (8). With the increasing global aging population, the number of sarcopenia cases is expected to reach 500 million by 2050 (9). The European Working Group on Sarcopenia in Older People 2(EWGSOP2) has identified muscle strength, muscle mass, and physical function as the main criteria for diagnosing sarcopenia (10). Despite being formally incorporated into the World Health Organization’s International Classification of Diseases (ICD) in 2016, sarcopenia may be underdiagnosed due to its insidious onset (11).

Sarcopenia increases the risk of falls, fractures, motor dysfunction, physical disability, and mortality. An observational study (12) showed no significant difference in the incidence of sarcopenia between the rotator cuff tears and normal control groups, indicating that sarcopenia cannot be used as a risk factor for rotator cuff tears. However, Kara et al. (13) obtained the opposite results and found that probable sarcopenia was a risk prediction factor for rotator cuff tears, which occurred in 18.6% of 1448 postmenopausal women. A relevant animal study has confirmed a correlation between sarcopenia and rotator cuff tears, which can occur before the onset of rotator cuff disease (14). However, due to the lack of consistency in the conclusions of existing studies, the causal relationship between sarcopenia and rotator cuff diseases is not clear. At the same time, based on case-control and cross-sectional observational studies, and considering the limitations of small sample size and insufficient follow-up, more powerful research designs are needed to further verify the causal relationship between sarcopenia and rotator cuff tears (15).

Mendelian randomization (MR) is a genetic analysis method (16) that uses single nucleotide polymorphisms (SNPs) as instrumental variables (IVs) to evaluate the causal relationships between exposure to relevant phenotypic characteristics and outcome factors. Due to the random allocation of individual genes at conception, resulting in the inheritance of a trait being independent of other traits and exhibiting randomness. Therefore, it is unlikely for the genotype of offspring to be influenced by lifestyle or environmental confounders (17). In comparison to observational studies, MR can more effectively mitigate confounding variables and reverse causality (18). With advancements in biological genetics, genome-wide association studies (GWAS) utilizing large sample sizes have identified multiple gene loci represented by SNPs that are closely associated with sarcopenia-related muscle characteristics, providing a valuable basis for causal investigation (19).

As a recently proposed disease concept, it is challenging to locate GWAS summary data for sarcopenia. According to the EWGSOP2 consensus, low muscle mass and strength (specifically grip strength and physical function) serve as primary indicators for measuring sarcopenia. Therefore, we have selected appendicular lean mass (ALM), low grip strength, and walking speed as exposure phenotypes (19). Utilizing validated SNPs and publicly available GWAS datasets, MR analysis was employed to investigate the causal relationship between traits related to sarcopenia and the development of rotator cuff tears. This study aims to provide clinical evidence-based support for managing sarcopenia in individuals with rotator cuff tears.

## 2 Materials and methods

### 2.1 Study design

**Figure 1:**
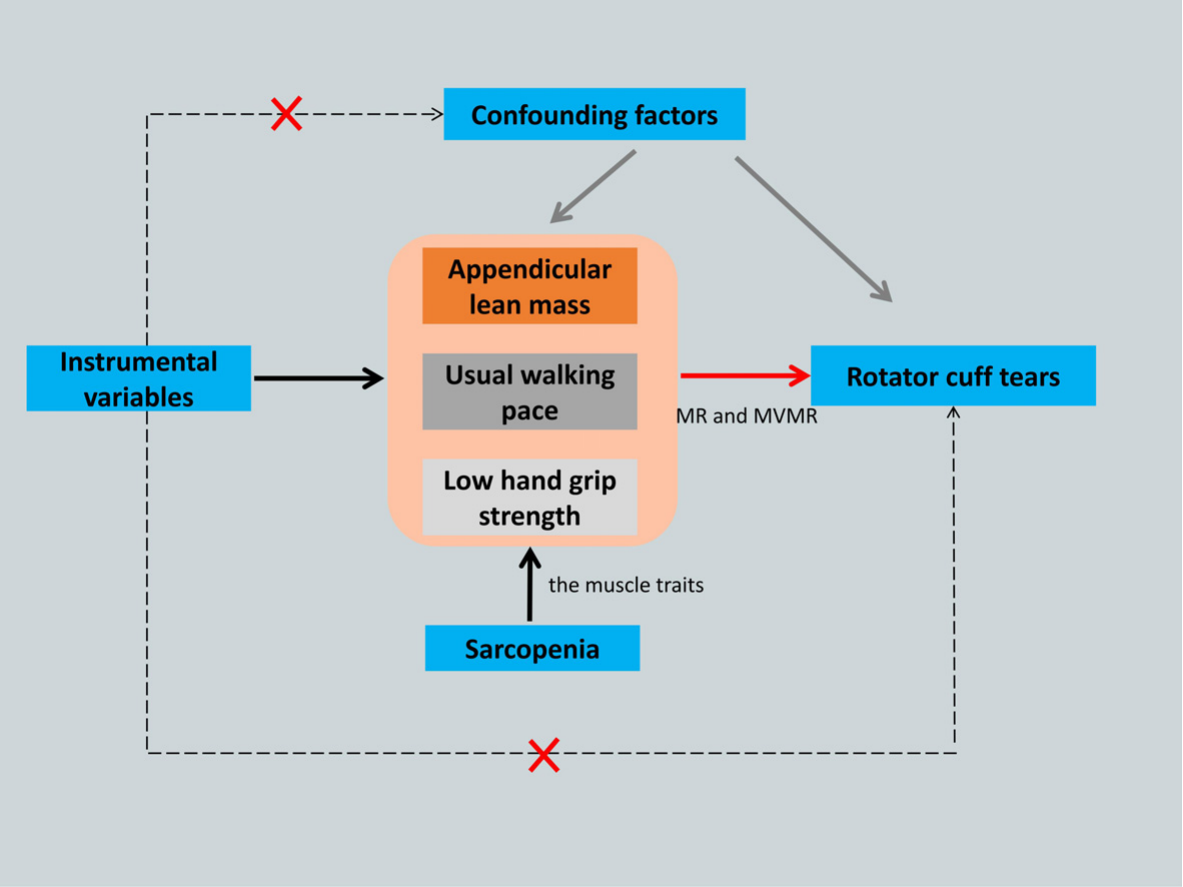
The framework flowchart of the Mendelian randomization study. Dotted arrow: Instrumental variables are not associated with any known or unknown confounding factors and influence rotator cuff tears not through any direct causal pathway. Black arrow: Instrumental variables reliably associated with the sarcopenia- related traits. Red arrow: Instrumental variables affect the rotator cuff tears only through appendicular lean mass, usual walking pace and low hand grip strength by using Mendelian randomization and Multivariate Mendelian randomization. MR, Mendelian randomization; MVMR, Multivariate Mendelian randomization.

MR requires three major assumptions (17): association, independence, and exclusion restriction, meaning that the SNPs used as IVs should have a robust association with the exposure factor and be independent of confounding factors, and that the outcome factor can only be influenced by the exposure factor. Multivariate MR (MVMR) analysis was used to evaluate the direct effects of various muscle characteristics on the causal relationship (20). The study flowchart is shown in **Figure 1**.

### 2.2 Data sources

To ensure the rigor and validity of the findings, we adhered to the following criteria in selecting the sarcopenia and rotator cuff tear datasets. Firstly, we selected the datasets covering disease phenotypes. Secondly, we screened statistics datasets from large-scale genetic biobanks and public summary data to ensure data undergo rigorous quality control and collation. Thirdly, we utilized the most up-to-date datasets to maintain the cutting-edge nature of our research. At the same time, datasets with a large sample size were preferable chosen to ensure the power of the statistical analysis and to reduce the potential for weak instrument bias.

All exposure and outcome genetic tools were obtained from different public gene datasets. The relevant characteristics of sarcopenia were obtained from the public gene database ‘IEU GWAS’ (https://gwas.mrcieu.ac.uk/datasets/), while the data on rotator cuff tears were obtained from the FinnGen database (https://www.finngen.fi). Detailed information for obtaining the exposure and outcome factors from GWAS is listed in **Table 1**. All data used were open to the public and did not require separate ethical reviews or informed consent.

**Table 1:** Data sources and description.

Phenotype	Consortium	Participants	Ancestry	GWAS ID	Year of publication
ALM^*^	UKB	450 243	European	ebi-a-GCST90000025	2020
Low hand grip strength	EWGSOP	48 596 cases and 207 927 controls	European	ebi-a-GCST90007526	2021
Usual walking pace	UKB	459 915	European	ukb-b-4711	2018
Rotator cuff tears	Finngen	26 376 cases and 299 606 controls	European	finn-b-M13_ROTATORCUFF	2023

*ALM, Appendicular lean mass.

Low skeletal muscle mass and strength (hand grip strength and physical function) are the primary indicators of sarcopenia, code ICD-10(M62.84) (11). The GWAS data for ALM were obtained from a pooled dataset of 450 243 participants aged 48-73 years (21). ALM was measured using bioelectrical impedance analysis. The low hand grip strength GWAS data were obtained from a pooled dataset of 256 523 participants (48 596 with low grip strength and 207 927 with normal grip strength) aged ≥60 years from the EWGSOP alliance (22). The maximum grip strength was recorded in kg, and low grip strength was defined as a grip strength of <30 kg in males and <20 kg in females. The GWAS data for walking speeds were obtained from the UK Biobank and included 459 915 participants of European ancestry with walking speed classified into three levels: slow, steady, or brisk (23).

We obtained summary data on SNPs related to rotator cuff tears as outcome factors from the FinnGen consortium database (26 376 cases and 299 606 non-cases), code ICD-10(M75.1). The FinnGen study is a large-scale genomics initiative that has analyzed over 500,000 Finnish biobank samples and correlated genetic variation with health data to understand disease mechanisms and predispositions (24). The FinnGen data used in this study were obtained from the whole-genome analysis results released in FinnGen R10, with participants having an average age of 55.57 years and an observation period spanning 1985 to 2022. Individuals with unclear sex and high missing/heterozygosity rates (>5%) were also excluded. To avoid population-specific bias, all participants were of European ancestry.

### 2.3 IV selection

To ensure the robustness and replicability of MR results, we selected SNPs that were significantly associated with the exposure factor from the pooled GWAS data as IVs (P <5×10^−8^). Removal of linkage disequilibrium (LD) reduced the non-random bias of IVs, and strict criteria were used to evaluate the independence of the SNPs (r^2^, 0.001; clump, 10 000kb) in order to select independent SNPs as IVs (25). The F-values of the selected IVs were calculated through univariate regression analysis for each IV and exposure phenotype (sarcopenia-related traits). The formula of calculating the IV’s F-statistic is as follows. IVs with an F-value ≤ 10 were eliminated to ensure sufficient explanatory power, thereby reducing the risk of weak instrumental variable bias (26).


$$R2=2^{*}{\left(1-MAF\right)}^{*}MAF^{*}\beta 2,$$

$$2F={\left(N-\kappa -1/\kappa \right)}^{*}(R2/1-R2)$$

### 2.4 Data analysis

All MR data analyses were conducted using the ‘TwoSampleMR’ (v.0.5.7), ‘MendelianRandomization (27) (v.0.9.0), ‘MRPRESSO’ (v.1.0), ‘MVMR (28)’ (v.0.4), and ‘forestplot’ (v.3.1.3) packages in R software (version 4.3.1; R Foundation for Statistical Computing, Vienna, Austria).

In MR, the inverse‐variance weighted (IVW) method is the main statistical analysis and can merge the Wald ratio estimates for each IV in the causal estimate (29). Both MR-Egger regression and the weighted median estimator (WME) were utilized for sensitivity analysis, to verify the reliability of the results (30, 31). The advantage of the weighted median method is that it can provide effective results, even if the number of effective IVs is reduced by at least half during the analysis. MR-Egger regression has the advantage of providing an effective causal effect evaluation, even when all SNPs are ineffective. We considered the consistent direction of the results from these three methods to indicate a relatively stable causal association. We used a forest plot to visualize the causal effect values of each IV and of the overall exposure on the outcome effect size.

We used the Cochran’s Q test to evaluate heterogeneity; *P <*0.05 indicated the presence of heterogeneity, and the IVW random-effects model was applied (32). Conversely, *P >*0.05 indicated no heterogeneity, and the IVW fixed-effects model was used. Funnel plots were used to visualize the results.

We used the MR-Egger regression intercept to conduct pleiotropy testing, ensuring that the selected IVs did not affect the outcomes through confounding factors (33). *P >*0.05 for the intercept indicated the absence of horizontal pleiotropy, while *P*<0.05 indicated that the causal relationship between the exposure and outcome did not hold. The MR pleiotropy residual sum and outlier (MR-PRESSO) test was used to detect outliers and correct the pleiotropy. Outliers were removed, and the remaining gene IVs were reanalyzed (34). We conducted a leave-one-out analysis of each SNP by gradually removing IVs to observe their direct impact on the results. We also used a function to graphically visualize the results.

Univariate MR was used to evaluate the “total effect” of sarcopenia-related traits on the causal relationship of rotator cuff tears. Then, the MVMR evaluated the “direct effect” of each trait on rotator cuff tears. *P*<0.05 indicated a statistically significant difference (20).

## 3 Results

### 3.1 IVs

Based on the selection criteria for IVs, we selected 690 SNPs with genome-wide significance as IVs for ALM, 16 for low hand grip strength, and 57 for usual walking pace. After removing palindromic sequences in the MR analysis, 536 SNPs were finally included for ALM, 14 for low hand grip strength, and 50 for usual walking pace. All F-values of the instrumental variables for the evaluation indicators of sarcopenia were >10, indicating that all three sets of IVs were strong indicators. The results are shown in **Supplementary Tables 1**-**3**.

### 3.2 MR analysis

**Figure 2:**
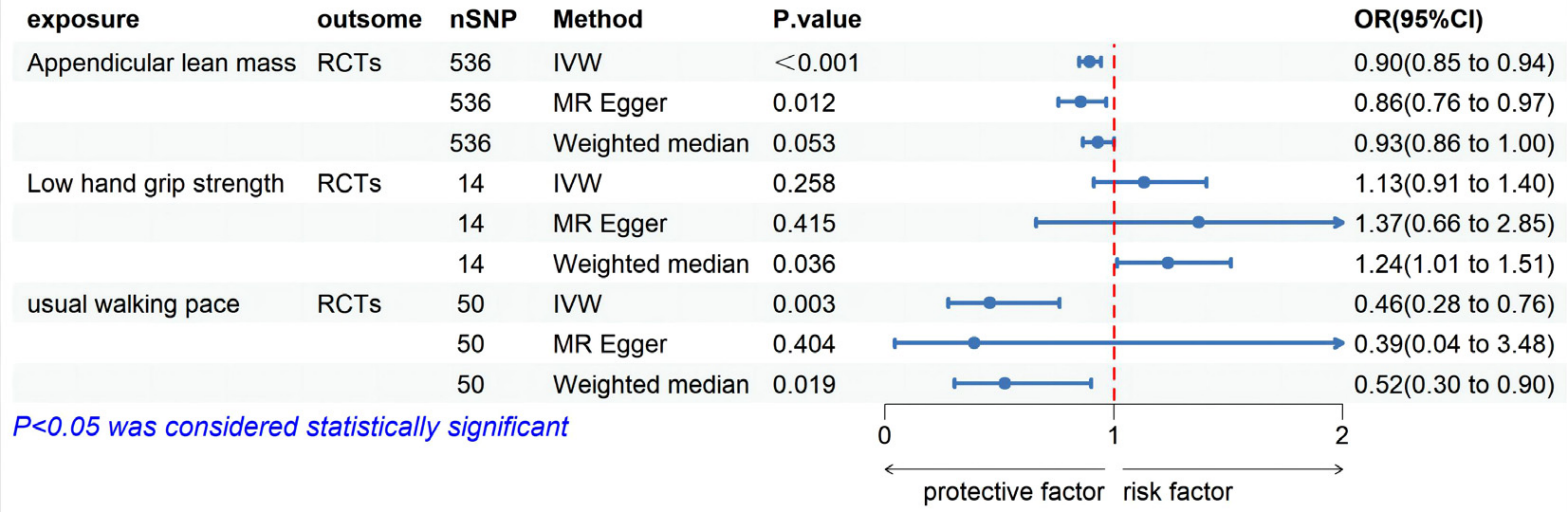
Forest plot of MR analysis estimates for the association between sarcopenia-related muscle traits and rotator cuff tears. OR, odds ratio; 95%CI, 95% confidence interval; IVW, inverse variance weighted random; RCTs, rotator cuff tears.

**Figure 3:**
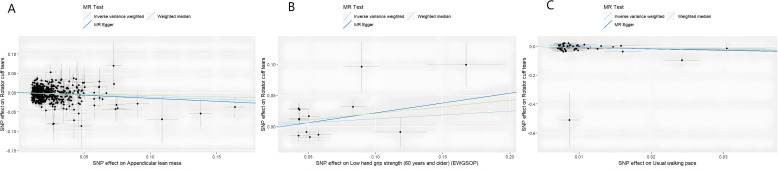
Scatter plots of univariate MR analysis. The horizontal coordinate is the effect of SNP on exposure, and the vertical coordinate is the effect of SNP on outcome. The horizontal and vertical lines on the dots are the confidence intervals of SNP for exposure effect and SNP for outcome effect, respectively. The colored lines correspond to the output of the MR analytical methods. **(A)** The causal association appendicular lean mass on rotator cuff tears. **(B)** The causal association of low hand grip strength on rotator cuff tears. **(C)** The causal association of usual walking pace on rotator cuff tears.

MR analysis based on the IVW method showed that genetically determined sarcopenia-related traits were causally associated with rotator cuff tears, as demonstrated in **Figure 2**. Scatter plots (**Figure 3**) were used to visualize the causal effect. ALM and usual walking pace were significantly associated with rotator cuff tears (odds ratio (OR) = 0.895, 95% confidence interval (CI), 0.758-0.966, *P*<0.001 and OR = 0.458, 95% CI, 0.276-0.762, *P* = 0.003, respectively), while there was no evidence to support a relationship between low hand grip strength and rotator cuff tears (OR = 1.132, 95% CI, 0.913-1.404, *P* = 0.26).

In the analysis of WME and MR-Egger, the beta value of sarcopenia muscle traits and rotator cuff tear was consistent with the IVW results. Moreover, the MR-Egger intercept term test of each trait was P>0.05, there was no horizontal pleiotropy, and the results were reliable (**Supplementary Table 4**).

Cochran’s Q heterogeneity test showed the presence of heterogeneity among the sarcopenia-related traits, which may be influenced by differences in the sample population or sequencing methods. Nevertheless, the results of the random-effects IVW method were reliable. The funnel plot exhibits a symmetric distribution (**Supplementary Figure 1**). Using the MR-PRESSO method, we found one outlier for ALM and low hand grip strength and two outliers for usual walking pace. After removing potential outliers, the random-effects IVW method remained stable (**Supplementary Table 4**).

The leave-one-out method was used to explore whether each SNP had a significant impact on the final result. Removing each SNP demonstrated that SNPs that did not introduce excessive bias to the results (**Supplementary Figure 2**).

In the MVMR analysis, genetic predictions of skeletal muscle mass and gait speed were found to have a causal effect on rotator cuff tears after excluding the influence of other exposure factors (OR = 0.918, 95% CI, 0.851-0.990, *P* = 0.03 and OR = 0.476, 95% CI, 0.304-0.746, *P =* 0.001, respectively) (**Figure 2**). However, the direct causal effect of the adjusted ALM on rotator cuff tears was reduced. Moreover, horizontal pleiotropy was not found in the multivariate analysis results (*P* = 0.10), which was consistent with the univariate analysis results (**Table 2**).

**Table 2:** Causal effects of sarcopenia-related traits on rotator cuff tears using MVMR^#^.

Exposure	nSNP^||^	MVMR Methods	beta	se**	P value	OR^†^(95%CI)^† †^	MR Egger p-intercept
ALM*	497	MVMR IVW^##^	-0.085	0.038	0.03	0.92 (0.85-0.99)	
	497	MVMR MR-Egger	-0.177	0.068	0.01	0.84 (0.73-0.96)	0.104
Low hand grip strength	497	MVMR IVW^##^	-0.029	0.056	0.61	0.97 (0.87-1.08)	
	497	MVMR MR-Egger	-0.037	0.057	0.51	0.96 (0.86-1.08)	0.104
Usual walking pace	497	MVMR IVW^##^	-0.743	0.229	0.001	0.48 (0.3-0.75)	
	497	MVMR MR-Egger	-0.805	0.232	0.001	0.45 (0.28-0.7)	0.104

^#^MVMR, Multivariate Mendelian randomization; || SNP, single nucleotide polymorphisms; **SE, standard error; ^†^OR, odds ratio; ^† †^95%CI, 95% conﬁdence interval; *ALM, Appendicular lean mass; ^##^IVW, inverse-variance weighted random.

## 4 Discussion

Research on the genetic causal association between sarcopenia and rotator cuff tears is lacking. In this study, we used MR analysis to analyze GWAS data, including single and joint effects, and found a causal relationship between sarcopenia and rotator cuff tears. The results showed that ALM and usual walking pace had a negative linear correlation with rotator cuff tears, which were protective factors for the latter. However, no evidence supported a genetic causal relationship between muscle strength and rotator cuff tears.

As a newly recognized muscle disease, sarcopenia was proposed in 2010. Initially, only skeletal muscle mass was used as the sole diagnostic criterion due to the lack of specific manifestations. With further clinical exploration, research on the correlation between sarcopenia and other musculoskeletal diseases has become more comprehensive. Han et al. (35) discovered that patients with sarcopenia exhibited a higher prevalence of shoulder pain, mucoid degeneration, and disordered muscle bundles in the supraspinatus tendon through musculoskeletal ultrasound comparison. Histopathological examination demonstrated varying degrees of fat infiltration and loss of muscle fibers, resulting in secondary strength loss and limb dysfunction (36). Chung et al. (37) compared the prevalence of sarcopenia between patients with rotator cuff injury and healthy individuals; they found that those with injuries had a higher likelihood of low muscle mass and strength which correlated with tendon tear size. The present study’s findings align with those results, suggesting a causal association between sarcopenia and rotator cuff tears where low muscle mass or physical function increases susceptibility to such injuries. Surprisingly though, this study did not establish a causal relationship between low grip strength and rotator cuff injuries. A prospective study examining grip strength before and after rotator cuff repair reached similar conclusions (38). Their founding indicated that grip strength was not related to shoulder dysfunction.

As key regulators of muscle homeostasis, fibro-adipogenic progenitors (FAPs) are widely distributed in skeletal muscle and tendinous tissues with bipotency (39). They play a crucial role in promoting the proliferation and differentiation of satellite cells, which are specific muscle stem cells, thereby enhancing skeletal muscle regeneration capacity and compensating for muscle fiber atrophy and functional decline (40). Additionally, FAPs exhibit inherent adipogenic and fibrogenic potential, leading to pathological aggregation. The expression of fibrogenic/adipogenic markers in FAPs was significantly elevated in the massive rotator cuff tear group, indicating an irreversible trend. Low muscle mass serves as the initial factor contributing to sarcopenia, characterized by varying degrees of fat infiltration and loss of type II muscle fibers (41). Studies have confirmed that mitochondrial dysfunction and immune response can induce skeletal muscle mass loss and disrupt muscular homeostasis. Mitochondria function as cellular energy communicators, regulating intracellular calcium concentration and cell proliferation, thereby playing a crucial role in muscle health, function, and homeostasis (42). In chronic muscle injury diseases, mitochondria can activate nuclear factor kappa B to trigger the NLRP3 inflammasome, which leads to the expression of tumor necrosis factor-α, interleukin (IL)-1β, and other inflammatory factors. This process enhances both local and systemic inflammatory responses and immune reactions while promoting ectopic fat accumulation (43). Consequently, it results in a decline in muscle function. IL-1β induces fibroblast activation along with the extracellular matrix of tendinous tissue. This chronic inflammatory response causes structural changes and disarrangement in tendons. Moreover, IL-1β interferes with the proliferation and adipogenic differentiation of FAPs (44). In addition to the effects of inflammatory factors on myofibers, mechanical stimulation also influences the number of FAPs. Exercise training has been shown to reduce muscle fiber loss (45).

Sarcopenia and rotator cuff tears are prevalent conditions among the elderly population. By investigating the causal association between sarcopenia and rotator cuff tears, have shown that sarcopenia-related muscle characteristics increasing the risk of rotator cuff tears. This study emphasizes the public health significance of restoring skeletal muscle mass and physical function in the prevention and acceleration of rehabilitation after rotator cuff tears. The functional repair of tendon and tendon-bone interface healing poses significant challenges in the treatment of rotator cuff tears. Although surgical or nonsurgical treatments (such as physical therapy and local or systemic pharmacotherapy) can restore normal anatomy and alleviate symptoms (46), 20%-25% of patients still experience tendon nonunion or progressive muscle atrophy, which significantly impacts their quality of life (47). Currently, there are numerous drug studies on sarcopenia; however, direct evidence supporting clinical efficacy and safety is lacking (48, 49). The primary treatment involves nutritional support combined with exercise intervention. For instance, elderly patients treated with whey protein supplements along with leucine and vitamin D have shown improvements in physical function (50). In addition to nutritional support, moderate intensity resistance exercise has been found to effectively enhance body composition and physical function (51). Relevant studies have also demonstrated that progressive resistance exercise or trunk block chain training for patients with rotator cuff tears can better alleviate pain caused by non-traumatic rotator cuff tears while influencing clinical outcomes positively (52, 53). However, no systematic study has reported on the effect of nutritional support on rotator cuff tears.

To our knowledge, this is the first MR study to investigate the association between sarcopenia and the risk of rotator cuff tears. Compared with the traditional observational studies, it has the advantages of reducing the risk of residual confounding and artificial bias. Our study highlights the predictive role of sarcopenia in the risk of rotator cuff tears and provides further insights and research directions to improve clinical recovery rates and optimize clinical disease management for rotator cuff tears. In addition, through sensitivity analysis and causal estimation, this study eliminated the biological effects of related risk factors such as lifestyle, age, and comorbidities on rotator cuff tears, verifying the robustness of the results. In the future, further exploration of the pathophysiological mechanisms involving bone, tendon-bone interface, and muscle tissue associated with sarcopenia and rotator cuff tears can be conducted, along with exploring therapeutic strategies for rotator cuff tears at a cellular or molecular level from the perspective of restoring skeletal muscle homeostasis. Additionally, increasing research on the combined intervention effects of exercise and nutritional support on outcomes of rotator cuff tear is warranted.

However, this study had some limitations. First, despite employing multiple analyses to ensure the robustness of the results, there may still exist unknown or unmeasured environmental and biological factors associated with sarcopenia or rotator cuff tears that could introduce confounding bias, such as exercise habits, external trauma factors and occupational exposure. Second, due to the utilization of the public gene datasets with the lack of sex- or age-stratified in this study, thereby failing to account for sex or age differences in the causal association between exposure and outcome. Nevertheless, as more GWAS studies are conducted with larger sample sizes, it becomes feasible to perform subgroup analyses based on age or sex. Third, The data of all associated exposure SNPs in the resulting GWAS were exclusively collected from European populations, thereby limiting their applicability in explaining the causal association of disease in Asian or other ethnic populations. Consequently, the causal association between sarcopenia and rotator cuff injury remains unknown in these non-European populations. Furthermore, selection bias may occur if those with a higher genetic liability to rotator cuff tears and a specific trait (e.g., higher upper extremity movements or the porter of physical activity) are more likely to participate in the study. This could induce an association between genetic liability for rotator cuff tears and the traits in our study. Finally, MR estimates lifelong rather than acute effects. Compared to short-term exposure, lifelong exposure usually has a greater impact on outcomes. This is because most exposures have a cumulative effect on the outcome over time; therefore, they cannot be extrapolated to study potential therapeutic effects in clinical settings. Our understanding of this topic remains limited, particularly regarding the age-related differences in disease development.

## 5 Conclusion

This MR study provides evidence of a genetic causal relationship between sarcopenia and rotator cuff tears. The evidence supports a negative correlation between ALM, usual walking pace, and the occurrence of rotator cuff tears. This confirms that sarcopenia-related muscle characteristics, including low skeletal muscle mass and physical function, may increase the risk of rotator cuff tears. However, no association was found between genetically predicted low hand grip strength and rotator cuff tears.

## Data Availability

The datasets presented in this study can be found in online repositories. The names of the repository/repositories and accession number(s) can be found below: https://gwas.mrcieu.ac.uk/datasets/ebi-a-GCST90000025/, ebi-a-GSCT90000025 https://ftp.ebi.ac.uk/pub/databases/gwas/summary_statistics/GCST90007001-GCST90008000/GCST90007526/, ebi-a-GCST90007526 https://gwas.mrcieu.ac.uk/datasets/ukb-4711/, Ukb-b-4711; https://r10.finngen.fi/pheno/M13_ROTATORCUFF, finn-b-M13_ROTATORCUFF.
