# Supplementary figures and images for: Causal relationship between sarcopenia and rotator cuff tears: a Mendelian randomization study
Source: Front Endocrinol (Lausanne). 2024 Oct 29;15:1436203. doi: 10.3389/fendo.2024.1436203 (PMC11555288; doi:10.3389/fendo.2024.1436203)

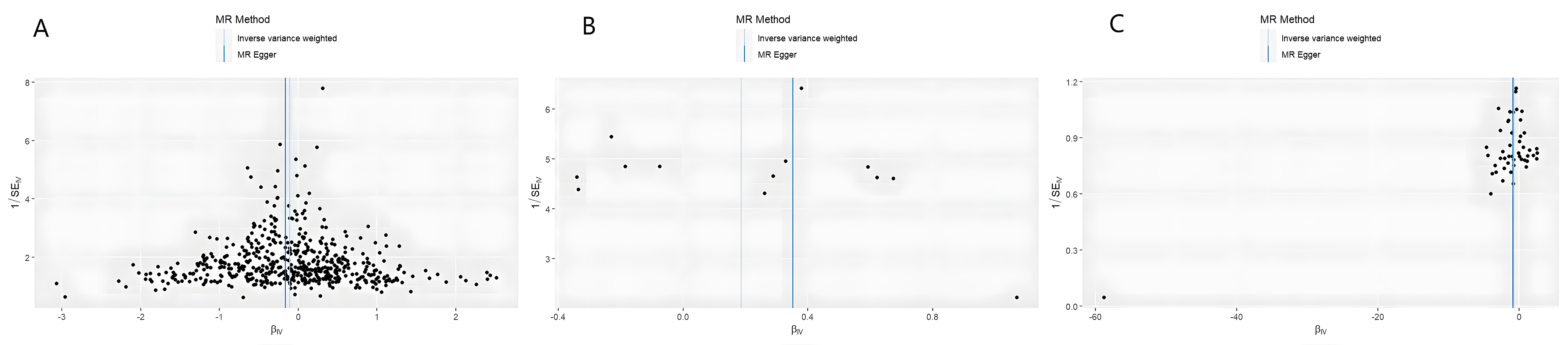

Supplement: Supplementary file 2 [file Image1.jpeg]

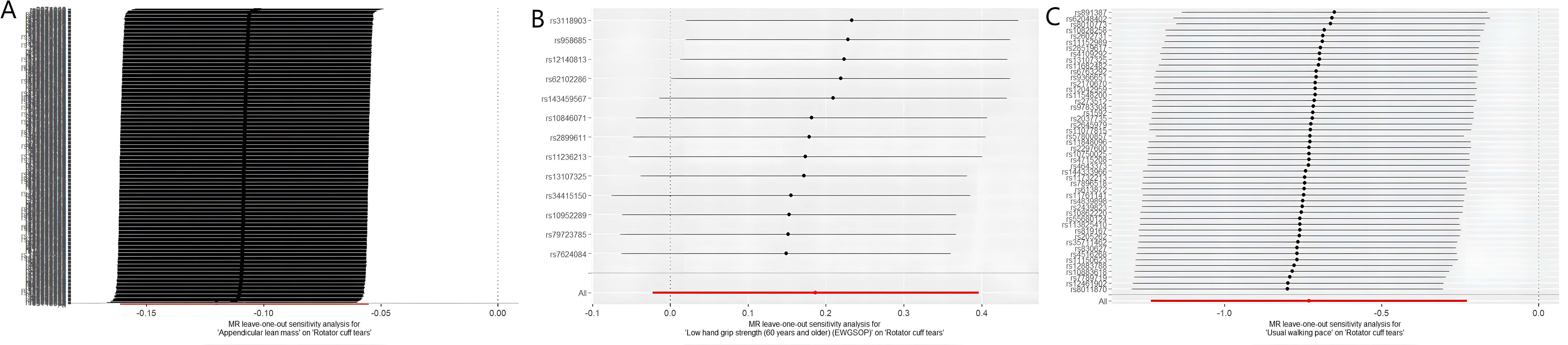

Supplement: Supplementary file 3 [file Image2.jpeg]
